# U.S. Funding Is Insufficient to Address the Human Health Impacts of and Public Health Responses to Climate Variability and Change

**DOI:** 10.1289/ehp.0800088

**Published:** 2009-02-27

**Authors:** Kristie L. Ebi, John Balbus, Patrick L. Kinney, Erin Lipp, David Mills, Marie S. O’Neill, Mark L. Wilson

**Affiliations:** 1 ESS, LLC, Alexandria, Virginia, USA; 2 Environmental Defense Fund, Washington, DC, USA; 3 Department of Environmental Health Sciences, Mailman School of Public Health, Columbia University, New York, New York, USA; 4 Department of Environmental Health Science, University of Georgia, Athens, Georgia, USA; 5 Stratus Consulting, Inc., Boulder, Colorado, USA; 6 Department of Epidemiology; 7 Department of Environmental Health Sciences and; 8 Department of Ecology and Evolutionary Biology, University of Michigan, Ann Arbor, Michigan, USA

**Keywords:** adaptation, climate change, health impacts, public health

## Abstract

**Background:**

The need to identify and try to prevent adverse health impacts of climate change has risen to the forefront of climate change policy debates and become a top priority of the public health community. Given the observed and projected changes in climate and weather patterns, their current and anticipated health impacts, and the significant degree of regulatory discussion underway in the U.S. government, it is reasonable to determine the extent of federal investment in research to understand, avoid, prepare for, and respond to the human health impacts of climate change in the United States.

**Objective:**

In this commentary we summarize the health risks of climate change in the United States and examine the extent of federal funding devoted to understanding, avoiding, preparing for, and responding to the human health risks of climate change.

**Discussion:**

Future climate change is projected to exacerbate various current health problems, including heat-related mortality, diarrheal diseases, and diseases associated with exposure to ozone and aeroallergens. Demographic trends and geophysical and socioeconomic factors could increase overall vulnerability. Despite these risks, extramural federal funding of climate change and health research is estimated to be < $3 million per year.

**Conclusions:**

Given the real risks that climate change poses for U.S. populations, the National Institutes of Health, Centers for Disease Control and Prevention, U.S. Environmental Protection Agency, and other agencies need to have robust intramural and extramural programs, with funding of > $200 million annually. Oversight of the size and priorities of these programs could be provided by a standing committee within the National Academy of Sciences.

Recent assessments have concluded that climate change presents real risks to human health and that the U.S. population will not be exempt from health impacts of recent and projected climate change (Confalonieri et al. 2007; Ebi et al. 2008). Given the observed and projected changes in climate and weather patterns and the significant degree of regulatory discussion under way in the U.S. government, it is reasonable to determine the extent of direct federal investment in research to understand and anticipate the human health impacts of climate change in the United States and worldwide. The need for this research has become more urgent given the significant degree of climate change to which the world is already committed. In addition, there is a need to assess the potential benefit and harm to human health from proposed policies to reduce greenhouse gas emissions. We were the authors of the chapter on human health for Synthesis and Assessment Product (SAP) 4.6, in *Analyses of the Effects of Global Change on Human Health and Welfare and Human Systems* (Ebi et al. 2008). We discovered during the process of researching and writing SAP 4.6 that federal investment in research on the health impacts of climate change has been extremely limited, leaving the United States insufficiently able to avoid, prepare for, and respond adequately to the risks.

We first review the key research needs related to climate change and health, using peer-reviewed publications to show that these research needs are not being met to a significant degree, then discuss steps that should be taken by the federal government to meet the research needs.

## Summary of Key Research Needs Related to Climate Change and Health in the United States

SAP 4.6 reviewed the scientific literature published since the first U.S. national assessment published in *Potential Health Impacts of Climate Variability and Change for the United States* (Patz et al. 2000) and reconfirmed that climate change poses a risk for U.S. populations, with uncertainties limiting the ability to quantify the projected number of increased injuries, illnesses, and deaths attributable to climate change (Ebi et al. 2008). Future climate change could exacerbate a number of current health problems, including heat-related mortality, diarrheal diseases, and diseases associated with exposure to ozone and aeroallergens. Demographic trends, such as a larger and older U.S. population, will increase overall vulnerability to these health risks; local geophysical and socioeconomic factors will influence vulnerability at the local level. In addition, the U.S. population may be at risk from climate-related diseases and disasters that occur outside U.S. borders, with travelers and refugees importing diseases not currently present. The unprecedented nature of climate change also may bring unanticipated consequences for public health.

Research on the health impacts of climate variability and change *a*) characterizes associations between weather/climate and health based on observed data; *b*) identifies observed effects of climate change on health; *c*) projects health impacts using models; or *d*) identifies, prioritizes, evaluates, implements, and monitors effective and timely response options (including adaptation and mitigation). Overall, the research base for understanding the health risks of climate change in the United States is limited, with most research exploring the associations between weather/climate and health (Table 1). The literature base on observed impacts of climate change contains only studies conducted outside the United States.

Given the range of impacts of climate change on health and the state of current research, SAP 4.6 recommended the following (Ebi et al. 2008):

- Improve characterization of exposure–response relationships, particularly at regional and local levels, including identifying thresholds and particularly vulnerable groups.
- Collect data on the early effects of changing weather patterns on climate-sensitive health outcomes.
- Collect and enhance long-term surveillance data on health issues of potential concern, including vectorborne and zoonotic diseases, air quality, pollen and mold counts, reporting of foodborne and waterborne diseases, morbidity due to temperature extremes, and mental health impacts from extreme weather events.
- Develop quantitative models of possible health impacts of climate change that can be used to explore the future consequences of a range of socioeconomic and climate scenarios. Such models will be essential for mid- to long-term planning.
- Increase understanding of the processes of adaptation, including social and behavioral dimensions, as well as the costs and benefits of interventions.
- Evaluate the implementation of adaptation measures. For example, evaluation of heat wave warning systems, especially as they become implemented on a wider scale, is needed to understand how to motivate appropriate behavior.
- Understand local- and regional-scale vulnerability and adaptive capacity to characterize the potential risks and the time horizon over which climate risks might arise. These assessments should include stakeholders to ensure that their needs are identified and addressed in subsequent research and adaptation activities.
- Improve comprehensive estimates of the co-benefits of adaptation and mitigation policies to clarify tradeoffs and synergies.
- Enhance collaboration across the multiple agencies and organizations with responsibility and research related to climate change-related health impacts, such as weather forecasting, air and water quality regulations, vector control programs, and disaster preparation and response.
- Anticipate infrastructure requirements needed to protect against extreme events such as heat waves and foodborne and waterborne diseases; to alter urban design to decrease heat islands; and to maintain drinking and wastewater treatment standards and source water and watershed protection.
- Develop downscaled climate projections at the local and regional scale to conduct the types of impact, vulnerability, and adaptation assessments that will enable adequate projections of and responses to climate change and to determine the potential for interactions between climate and other risk factors, including societal, environmental, and economic factors. The growing concern over impacts from extreme events demonstrates the importance of climate models that allow for stochastic generation of possible future events, to assess not only how disease and pathogen population dynamics might respond, but also whether levels of preparedness are likely to be adequate.

Realistically assessing the potential health effects of climate change must include consideration of the capacity to manage the impacts of new and changing climatic conditions. Individuals, communities, governments, and other organizations currently engage in a wide range of actions to identify and prevent adverse health outcomes associated with weather and climate such as heat waves, wild-fires, hurricanes. Although these actions are generally viewed as having been largely successful historically, two recent surveys suggest that climate change will challenge the ability of current programs and activities to control climate-sensitive health determinants and outcomes (Balbus et al. 2008; Maibach et al. 2008; Wells Bedsworth 2008). Although some level of preparedness exists, there is a long way to go before the country’s adaptive capacity is at a sufficient level. The prepared-ness gap includes not just infrastructure and capacity, but also fundamental knowledge and the availability of reliable decision support tools. Preventing additional morbidity and mortality will require modification of current and implementation of new programs and activities to increase resilience to climate change, taking into consideration the local context, including socioeconomic, geographic, and other factors. Research is needed to identify effective and efficient programs and activities, as well as how to transfer lessons learned to other communities to assure broad protection of public health (Ebi et al. 2008).

An issue not specifically highlighted in the research recommendations is the increasing need for multidisciplinary research that addresses the interactions of impacts across sectors (for example, decreasing precipitation leading to reduced freshwater availability, thus increasing the potential for foodborne and waterborne diseases, or how changes in temperature and precipitation affect land use, which could affect the geographic spread and intensity of transmission of a range of vectorborne diseases). The possible mental health impacts of climate change, nutritional issues related to food scarcity, and population displacement are other issues requiring further research. Also not included in the list of research needs is the importance of identifying how to communicate most effectively the health risks of climate change, and the possible health harms and benefits of adaptation and mitigation options to address these risks, to motivate appropriate responses across all sectors of society. The possible health harms and benefits from mitigation technologies and policies were explicitly excluded from consideration in SAP 4.6 and are critically important to understand and better inform policy development.

The *Fourth Assessment Report of the Intergovernmental Panel on Climate Change* and other international assessments identified similar research needs, primarily focused on needs outside the United States (Confalonieri et al. 2007). Several areas of concern, such as the geographic spread of human vectors and pathogens, can represent new and emerging risks to U.S. populations.

## Progress in Addressing Key Research Needs Related to Climate Change and Health in the United States

Quantifying the current level of U.S. funding of climate and health research raises the issue of which programs and projects should be included in the tally. Because the information on the research conducted by intramural programs is often not publicly available, the budgetary costs of the valuable research conducted intramurally by scientists at U.S. agencies are not included in the calculations, which focus only on extramural funding.

Two general approaches to estimating research investments are to count all programs sponsoring research that is in some way related to health and climate, or to count only programs sponsoring research that is specifically directed at climate change impacts on health. Estimates of federal funding of the health impacts of climate change have generally taken the first approach.

## Federal Research on Climate Change and Health

When the U.S. Global Change Research Program [U.S. GCRP; since renamed the Climate Change Science Program (CCSP)] started in 1989, human health was included in the topic area of human interactions. Health studies were mentioned as a high priority need in the U.S. GCRP 1990 annual report, *Our Changing Planet*, but the health problem identified was ultraviolet (UV) radiation exposure, which is related to stratospheric ozone depletion and not climate change (U.S. GCRP 1990). There was no mention of funding for specific health studies. By 1996, *Our Changing Planet* had relabeled the topic area as “human dimensions” and again listed climate change and health studies as a priority (U.S. GCRP 1996). Roughly $28 million was reported financed by the National Institutes of Health (NIH) for the study of UV radiation. The National Oceanographic and Atmospheric Administration (NOAA) and the U.S. Environmental Protection Agency (EPA) funded modest research on human dimensions and regional vulnerabilities, respectively, but there was no explicit mention of supporting human health research.

Beginning in fiscal year (FY) 1999, the U.S. EPA began to explicitly study interactions between climate change and human health as part of a new initiative on the consequences of climate change. In FY 2000, the U.S. EPA, NOAA, National Science Foundation, National Aeronautics and Space Administration, and Electric Power Research Institute established a Joint Announcement on Climate Variability and Human Health to develop and demonstrate the feasibility of new approaches to investigate and develop tools to integrate useful climate information into public health policy and decision making. This Joint Announcement had a funding level of approximately $1.5 million per year. The program ended in 2005.

The National Research Council (NRC), in its review titled *Evaluating Progress of the U.S. Climate Change Science Program: Methods and Preliminary Results* (NRC 2007), concluded the following:

This inquiry showed that few agency programs are aimed explicitly at human contributions and responses research, so detailed estimates of expenditures could not be generated. Relevant research may or may not be counted as CCSP, and some research that is clearly peripheral to research element objectives is included in the program accounts. For example, the National Institutes of Health (NIH) program on health effects of stratospheric ozone constitutes more than two-thirds of the reported human contributions and responses budget, yet it is only tangentially concerned with climate change or social science research. Another large fraction of the funding goes to decision support activities, most of which lack a human dimensions research component (see Chapter 5). Including such programs paints a distorted picture of CCSP human contributions and responses research. Funding for human dimensions research is likely on the order of $25 million to $30 million per year, excluding NIH research on the health effects of ozone and National Aeronautics and Space Administration (NASA) decision support activities (Appendix B).

This includes all human dimensions research, not just the health impacts of climate change.

Further, in response to the question “What are the potential human health effects of global environmental change, and what climate, socioeconomic, and environmental information is needed to assess the cumulative risk to health from these effects?” the NRC (2007) review concluded that

The vast bulk of this research program involves either health effects of ultraviolet radiation or satellite measurement of particulate matter concentrations for health-related analysis.

The report concluded that the CCSP lags in understanding the human health impacts of climate change. Further, efforts to understand climate change impacts on society, to analyze mitigation and adaptation strategies, and to study regional impacts are “relatively immature.” It recommended that the CCSP adjust the balance between climate science and application. That rebalancing has yet to take place.

Consistent with the NRC (2007) review, one conclusion from Ebi et al. (2008) was that

Few research and data gaps have been filled since the First National Assessment. An important shift in perspective that occurred since the First National Assessment is a greater appreciation of the complex pathways and relationships through which weather and climate affect health, and the understanding that many social and behavioral factors will influence disease risks and patterns (NRC 2001). Several research gaps identified in the First National Assessment have been partially filled by studies that address the differential effects of temperature extremes by community, demographic, and biological characteristics; that improve our understanding of exposure-response relationships for extreme heat; and that project the public health burden posed by climate-related changes in heat-waves and air quality. Despite these advances, the body of literature remains small, limiting quantitative projections of future impacts.

## Recent Levels of U.S. Funding of Research on the Health Impacts of and Public Health Responses to Climate Variability and Change

Two authors of the present commentary (K.L.E. and J.B.) testified on 10 April 2008 before the Senate Committee on Health, Education, Labor and Pensions on the potential health impacts of climate change. On the basis of SAP 4.6, Ebi testified that “A severe limitation to understanding current and projecting future health impacts of climate change in the U.S. is the very low level of research aimed at providing quantitative projections of the number of increased injuries, illnesses, and deaths that could be attributable to climate change.” In follow-up questions for the record, Ebi and Balbus were asked: “According to their own estimates, NIH spends $164 million each year on the health effects of climate change, significantly more than they spend on autism, a disorder that affects millions of children today. CDC [Centers for Disease Control and Prevention] spends additional money on climate change through their National Center on Environmental Health. It seems to me that there is a pretty good federal funding effort going on. However, you indicated in your testimony that more funding for research was needed. Could you estimate how much more would be needed?”

To be considered as research to address the health risks of climate change, such research should, at a minimum, analyze associations between weather/climate variables and climate-sensitive health outcomes using empirical data; identify observable health impacts of climate change; project impacts under a range of climate and socioeconomic scenarios; or identify and evaluate response options (including barriers to implementation).

Sponsoring research on associations between asthma and air pollutants, for example, does not provide specific information on how, if at all, climate change could affect the incidence and severity of asthma (including where and when, and who is most at risk), the best options for reducing projected increases, and the associated health care and other costs. Similar examples could be provided for the other health outcomes of concern. When West Nile virus was introduced into the United States, agencies did not increase their general funding of vectorborne disease research (or highlight that research as the appropriate response to the problem), but rather established directed programs intended to answer specific questions relevant to the threat of West Nile virus. Without programs directed specifically at the unique challenges posed by changes in climate-related factors, identification and management of climate change related health risks will be inadequate.

With regard to the estimated NIH annual funding of $164 million, none of the studies projecting the health impacts of climate change cited in SAP 4.6 acknowledged NIH funding, a requirement for research conducted with NIH support. Only one study of the associations between weather/climate and health acknowledged partial NIH funding (Naumova et al. 2006). This indicates that NIH is not directing a total of $164 million in funding to climate change and health research.

According to the information provided by the Department of Health and Human Services to the CCSP on the appropriations requests relevant to CCSP goals for FY 2009, approximately $46.8 million in research will be funded. This is about 3.6% of the $1,309 million reported by CCSP as the federal budget on climate change research (U.S. CCSP 2008). All of that funding is directed at the health impacts of UV radiation, conducted by the National Cancer Institute ($31.4 million), the National Institute of Environmental Health Sciences (NIEHS) ($13.7 million), and the National Institute of Arthritis and Musculoskeletal and Skin Diseases ($1.7 million). As previously noted, UV radiation and associated health outcomes are not clearly related to climate change. Funding in prior years was slightly higher but did little to improve our understanding of the health risks of climate change.

Most climate change–related health risks of current concern are of importance irrespective of climate change. Heat waves, storms, wildfires, aeroallergens, and ozone currently cause adverse health impacts in the United States, and every year people experience and occasionally die from vector-, food-, and water-borne diseases. Therefore, research to better understand the factors other than climate that affect the incidence and geographic range of these health impacts is relevant, but not sufficient, for preparing for the health risks of climate change. Apparently counting all grants for climate-sensitive health outcomes, whether or not the research is directed at understanding the risks of and responses to climate change, Sharon Hrynkow, Associate Director of NIEHS, estimated that NIEHS funds approximately $100 million annually in research related to climate change (NIEHS 2008). A search of the NIEHS Web site identified no call for proposals on climate change and health and identified no extramural research under the key words “climate” and “change.”

A search of the Web site of the NIH Office of Extramural Research using the search term “climate change” identified two funding announcements: one on emerging infectious diseases and one on behavioral and social research on disasters and health. Both were released in 2006. The announcements suggested that climate change may be a risk factor for emerging infectious diseases or may increase the risks of weather-related disasters, but the proposals were not required to address the health risks of climate change. A search of the NIH computer database for retrieving information on scientific projects (NIH 2008) for the years 2002–2008 for “climate” and “change” retrieved 144 hits; these hits were not unique (i.e., there were multiple hits for the same project). The projects identified addressed issues related to tobacco, HIV/AIDS, obesity, mental health, autism, and drug abuse, as well as projects to model tsetse fly simulation models and to understand causes of harmful algal blooms and *Vibrio cholerae*. On the basis of this evidence, we assert that the NIH budget actually devoted to direct research on the health risks of and public health responses to climate change is considerably less than $164 million.

In addition to NIH and NIEHS programs, an effort to quantify federal climate and health funding should consider resources expended by the CDC, U.S. EPA, and other agencies that seek to understand and prevent climate-sensitive health outcomes, such as disease surveillance programs, programs to ensure safe food, water, and air, etc.

Citing urgent threats including climate change, CDC Director Julie Gerberding advocated in March 2008 for an increase in CDC funding. Instead, the President’s FY 2008 budget cut CDC funding by 2.8% from what would have maintained 2007 funding levels adjusted for inflation. The proposed FY 2009 budget will cut CDC funding further. The CDC Climate Change Policy states “the public health effects of climate change remain largely unaddressed” (CDC 2007).

The CDC’s National Center on Environmental Health has run a series of workshops on the health risks of climate change since January 2007. These workshops included efforts to solicit from researchers and affected communities recommendations on research directions for the CDC. The CDC is currently funding well under $1 million in research on heat-related morbidity and mortality that is relevant to climate change (Frumkin HF, McGeehin MA, personal communication). A new solicitation intends to fund $3 million in FY 2009 on the impacts of climate change on human health.

The only agency consistently funding research on the health impacts of climate change has been the U.S. EPA, primarily through the Science to Achieve Results (STAR) program. A search of the National Center for Environmental Research Web site indicates that two solicitations on climate change and health in 2005 funded projects on decision support systems for public health and on the impacts of climate variability and change; for the latter solicitation, three STAR grants were funded for a total of nearly $2.5 million over several years. Before that, the National Center for Environmental Assessment made several awards through an interagency collaboration, but only a small proportion of these awards were focused on health. One solicitation on air quality (not necessarily estimating health impacts) was funded in 2006. Two solicitations were released in 2008 on the impacts of climate change on allergic airway disease and on adaptation for future air quality analysis and decision support tools in light of global change impacts and mitigation. Total funding of approximately $6.8 million is anticipated over 3–4 years.

## Actions to Address Key Research Needs Related to Climate Change and Health in the United States

Effectively addressing the health risks of climate variability and change will require wide-ranging responses from federal and state agencies and departments. Because the health risks of and public health responses to climate change cover a broad range of issues, and because the risk and responses will change over temporal and spatial scales, there should be federal coordination of programs and activities, within the CCSP or a similar organization, to ensure that funding focuses on critical research needs to address current gaps and those likely to arise within the next few decades. Programs and activities designed to address climate change and health issues should be established within all federal agencies whose missions mandate human health, including Departments of Commerce (specifically NOAA), Health and Human Services (particularly CDC), Homeland Security, the U.S. EPA, NIH, National Science Foundation, and U.S. Geological Survey.

A robust research strategy to address the health risks of climate change, including the health aspects of climate mitigation and adaptation policies, should integrate the four broad research activities mentioned previously: characterizing associations between weather/climate and health based on observed data; identifying observed effects of climate change on health; projecting health impacts using models; and identifying, prioritizing, evaluating, implementing, and monitoring effective and timely response options (including adaptation and mitigation). Key public health research categories that address these essential services include surveillance and monitoring; field, laboratory, and epidemiologic research; model development; development of decision support tools; and education and capacity building of the public and public health and health care professionals (Frumkin et al. 2008).

Other initiatives can provide benchmarks for recommendations of the size of federal programs on climate change and health. One initiative of relevance is the federal research program on particulate matter (PM). In 1997, President Clinton called for a partnership of federal agencies to develop a coordinated inter-agency research program on PM (NRC 1998). In 1998, at the request of the U.S. EPA, a committee of the NRC assessed the state of research on PM and additional research needs, laying out a 13-year research agenda and recommended budget, calling for the U.S. EPA to spend $40–60 million annually for the first 6 years, with amounts declining to $15 million in 2015 (NRC 1998). The NRC noted explicitly that these amounts are not intended to represent the recommended total PM research budget for the U.S. EPA or the nation. Actual annual funding generally followed the recommendations for the first 6 years (NRC 2004).

A comprehensive surveillance and monitoring system to address the health risks of climate change that included indicators for climate, atmospheric, and ecosystem conditions as well as the health of domestic animals, wild animals, and humans would provide the information needed to implement timely and appropriate programs and activities to reduce the health risks of climate change, as well as the data needed for other research and modeling. A comprehensive surveillance and monitoring system would require expansion and greatly heightened cooperation among existing programs within the USDA, NASA, U.S. EPA, and CDC. Costs would need to be determined by the individual agencies but would likely exceed $100 million annually.

Field, laboratory, and epidemiologic research programs to understand the role of weather and climate in the geographic distribution and incidence of climate-sensitive health outcomes, possible thresholds, and the design of decision support tools should expand on current intramural programs and multiagency extramural grants, as well as building new capacity for this research through centers of excellence. These programs should focus primarily on the interactions between climate variability/change and human health and would not replace existing research programs on various aspects of climate-sensitive health problems, such as studies of air pollution and asthma. Regional centers of excellence should be selected based on geographic representativeness and expertise in locally relevant health issues. These centers would work in consultation and cooperation with local officials and affected communities to design research programs and specific interventions. Given the range of geographic and health issues, at least 10 centers would be necessary. A conservative estimate of needed funding is $5 million annually for each center, with an additional $100 million annually for intramural and extramural investigator-initiated research.

The health sector currently has no model (software tool) that can be used at the national, state, and regional levels to project the health risks of climate change. Developing such a model has been identified as a high priority by state and local public health officials. Approximately $1–2 million would be needed for initial model development. Additional funding would be required to integrate and link this with models of water resources, agriculture, and the like, thereby providing more comprehensive insights into the risks of climate change and the possible consequences of policy choices.

## Conclusions

There are considerable uncertainties as to how much relevant and directed research the federal government is funding to address the health risks of and public health responses to climate variability and change. More clarity and transparency in research funding, both extramural and intramural, is clearly needed (Government Accounting Office 2006). In capturing that information, categories of climate change–related research need to be at fine enough scales to accurately gauge how much funding is directed to human health research, not just all human dimensions. Further, it will be important to understand that focus of research activities to ensure that all priority areas are being adequately addressed.

Based on data available from agency Web sites and excluding associated studies, it appears that current federal funding directly addressing the health risks of climate change is approximately $3 million annually; this number would be approximately $1 million without two recent solicitations from the U.S. EPA. A new solicitation from the CDC also intends to fund $3 million in FY 2009. These estimates are significantly less than funding figures provided to CCSP and are inadequate to address the real risks that climate change poses for U.S. populations.

This inadequate level of U.S. funding appears to be attributable to the low priority placed on identifying and managing the health risks of climate change by Congress and the federal government. There are five overarching goals for CCSP for FY 2009 (U.S. CCSP 2008). Two are relevant to human health. The fourth, Theme 4, is to understand the sensitivity and adaptability of different natural and managed ecosystems and human systems to climate and related global changes. However, the three identified focus areas do not explicitly mention human health as a priority. These focus areas are intended to *a* ) improve knowledge of the sensitivity of ecosystems and economic sectors to global climate variability and change, *b*) identify and provide scientific inputs for evaluating adaptation options, in cooperation with mission-oriented agencies and other resource managers, and *c*) improve understanding of how changes in ecosystems (including managed ecosystems such as croplands) and human infrastructure interact over long time periods. Theme 5 is to explore the uses and identify the limits of evolving knowledge to manage the risks and opportunities related to climate variability and change. Again, the identified focus areas do not spotlight human health (support informed public discussion of issues of particular importance to U.S. decisions by conducting research and providing scientific synthesis and assessment reports; support adaptive management and planning for resources and physical infrastructure sensitive to climate variability and change; build new partnerships with public and private sector entities that can benefit both research and decision making; and support policy making by conducting comparative analyses and evaluations of the socioeconomic and environmental consequences of response options). Although some of these focus areas can be applied to human health issues, it is essential that addressing the health risks of climate change be explicitly mentioned in CCSP goals.

More important, given the current and projected health risks of climate change in the United States, Congress needs to allocate funds to federal agencies whose mission mandates include human health; these agencies should maintain and enhance programs (and appropriate funding) to specifically address climate change risks in a timely and efficient manner. Based on a simple assessment of the research needs, the level of federal funding directed at climate change and health research should be > $200 million annually.

This suggested level of effort must rely on continued robust programs of research relevant to climate change and health. Some of this research is directed at better understanding the relationships between climate change and the intermediate drivers of human health. For example, the U.S. EPA Global Change Research Program recently completed a 9-year-long assessment of the implications of climate change for regional air quality (U.S. GCRP 2009). This work has clear implications for projections of health impacts of air pollution in a setting of climate change. Other research, directed at gaining a better understanding of nonclimate drivers of human diseases, is also necessary to inform analyses and projections of climate impacts. For example, the NIEHS and CDC have conducted extensive research on asthma, vectorborne diseases, and other climate-sensitive health outcomes that is required for understanding and predicting weather/climate exposure response relationships and developing effective and timely adaptation and mitigation options.

Climate change is not a pollutant in the classical sense used in public health; it is projected to fundamentally alter the natural and humanmade systems on which our society relies, including air, water, agriculture, and ecosystems. Responses to climate change may alter energy, transportation, and other systems required for our societies to function. The health risks of climate change may arise from changes in any of these systems. Better understanding is needed of the interactions of these systems with health, including risks and opportunities for interventions to improve population health. Ensuring that a federal research program prepares the United States for the current and projected health impacts of climate change would be facilitated by establishing a standing committee within the National Academy of Sciences to advise on the size, priorities, and balance of such a program, through independent and regular evaluations of the state of knowledge and critical research gaps to address current and projected health risks.

The research programs advocated in this commentary are not primarily of an exploratory, academic nature. Much of the recommended research on climate change and health is targeted, focused research and data collection needed to design and implement timely and appropriate preventive actions. In the context of a national economic crisis, it is important to note that funding of climate change and health research directly linked to protective action at the local level is a wise investment consistent with the goals of restoring economic stability, justice, and environmental quality, and reducing health care costs. For example, a nonprofit organization, ICLEI (International Council for Local Environmental Initiatives)–Local Governments for Sustainability, is demonstrating that local governments and communities can save hundreds of thousands of dollars through energy efficiency measures, many of which contribute to better air quality in addition to economic benefits (ICLEI 2007). Public health improvements in general should also support good stewardship of natural and financial resources through reductions in energy consumption and greenhouse gas emissions. The costs of investing in climate change and health research will be offset by reduced health care costs resulting from improved public health preparedness and optimization of mitigation and adaptation policies.

Evidence is accumulating that climate change is adversely affecting human health in other parts of the world (e.g., Confalonieri et al. 2007). The lack of attention from the federal government on the health risks of climate change to U.S. populations is needlessly putting multitudes at risk.

## Figures and Tables

**Table 1:** Relative number of studies addressing the health risks of climate change in the United States.

Health outcome	Studies exploring associations with weather/climate	Studies projecting the health impacts of climate change
Heat waves	++	+
Other extreme events	+	0
Waterborne and foodborne diseases	++	0
Vectorborne and zoonotic diseases	+	0
Air pollution (limited areas)	++	+
Aeroallergens	+	0
Other health impacts including mental health, nutritional issues related to food security, and population displacement	+	0

+, a few published studies; ++, a relatively larger number of published studies.
